# Maternal early life trauma and wheeze in young children: could there be an association?

**DOI:** 10.1186/1710-1492-10-S1-A45

**Published:** 2014-03-03

**Authors:** Alicia N Pawlowski, Anita L Kozyrskyj, Suzanne C Tough, Sandra A Wiebe, Lionel J Dibden

**Affiliations:** 1 Pediatrics, University of Alberta, Edmonton, AB, Canada, T6T 1C9; 2 Pediatrics, University of Calgary, Calgary, AB, Canada, T3B 6A8

## Background

Chronic trauma in childhood can program an abnormal stress reaction in affected children, resulting in lifelong difficulties with stress management and poor health outcomes linked to changes in the immune system. At the same time, maternal stress during pregnancy and the postpartum period has been linked to a number of diseases in childhood, including wheeze and asthma. Given the potential for her own maltreatment in childhood to shape a mother’s later response to stress during pregnancy, it seems plausible that children may demonstrate inheritance of their mother’s childhood trauma through their own health issues. We hypothesize that preschool children are more likely to have a wheeze or allergic disorder if their mother has a history of childhood abuse, independent of her distress during pregnancy and postnatally.

## Methods

The Community Perinatal Care (CPC) Study of Calgary provides extensive data on 791 medically low risk mothers, of whom 61 (7.7%) and 77 (9.7%) had children with a wheezing disorder and allergies, respectively, at age 3. In order to investigate how past maternal maltreatment might be associated with wheeze and asthma in young children, a number of validated questionnaires within the CPC study were used to measure and categorize past maternal trauma . The abuse variables produced were used in logistic regression models, adjusted for relevant confounding factors, to determine their association with the development of wheeze or allergies in preschool children.

## Results

Calgary women fell within reported Canadian norms in their experience of childhood maltreatment, as did their children in their reports of wheeze and allergy. After adjustment, multiple logistic regression revealed associations between different maternal childhood abuse types and wheeze or allergies at age 3. There was a significant association between a mother’s experience of household dysfunction before age 5 (defined as having parents who fought frequently and violently, and at least one parent who had a substance abuse problem) with childhood wheeze (adjusted OR: 5.01, 95%CI: 1.41-17.84). Given sex interactions of a moderate strength, we decided to also perform an analysis centered on gender. In women who were sexually abused before age 8, their sons were more likely to have allergies (adjusted OR: 2.96, 95%CI: 1.09-8.08). Experiencing more than 2 types of maternal childhood abuse before age 5 increased the likelihood of daughters having a wheeze disorder (OR: 6.92, 95% CI: 1.39-34.49).

## Conclusions

Stressful maternal childhood experiences are associated with the development of wheeze and allergy in children.

